# Resilience, sleep quality and sleepiness in Peruvian medical students: a multicenter study

**DOI:** 10.3389/fpsyt.2024.1284716

**Published:** 2024-08-15

**Authors:** Jean Pierre Zila-Velasque, Pamela Grados-Espinoza, Brenda Sofia-Caira Chuquineyra, Mills Diaz-Vargas, Gabriela Stefanie Sierra Calderón, Sthefanny Choquegonza, Mario S. Temoche-Rivas, Karina Siguas Peixoto, Mario J. Valladares-Garrido, Virgilio E. Failoc-Rojas

**Affiliations:** ^1^ Red Latinoamericana de Medicina en la Altitud e Investigación (REDLAMAI), Pasco, Peru; ^2^ Facultad de Medicina Humana, Universidad Nacional de San Agustín, Arequipa, Peru; ^3^ Facultad de Medicina Humana, Universidad Nacional del Centro del Peru, Huancayo, Peru; ^4^ Facultad de Ciencias de la Salud, Escuela Académico Profesional (EAP): Medicina Humana, Universidad Continental, Huancayo, Peru; ^5^ Facultad de Medicina Humana, Universidad Privada de Tacna, Tacna, Peru; ^6^ Facultad de Medicina Humana, Universidad Nacional de Piura, Piura, Peru; ^7^ Facultad de Medicina “Rafael Donayre Rojas”, Universidad Nacional de la Amazonía Peruana, Iquitos, Peru; ^8^ Facultad de Medicina, Universidad Continental, Lima, Peru; ^9^ Servicio de Epidemiología, Hospital Regional Lambayeque, Lambayeque, Peru; ^10^ Vicerrectorado de Investigación, Universidad San Ignacio de Loyola, Lima, Peru

**Keywords:** resilience, sleep-wake disorders, sleepiness, medical students, Peru

## Abstract

**Background:**

Resilience can mitigate the negative impact produced by the COVID-19 pandemic. Medical students endure significant academic stress, so adjusting to sudden changes can present greater mental health challenges. The aim is to identify the level and prevalence of resilience and to know what are the educational variables and is sleep quality associated with resilience.

**Methods:**

A cross-sectional study was conducted using an online questionnaire. The survey was elaborated in Google Forms and shared through social networks. The outcome was resilience, measured with the Connor Davidson Resilience Scale. Its association was assessed with sleep quality (measured with the Pittsburgh Sleep Quality Index), daytime sleepiness (measured with the Epworth Sleepiness Scale), and selected academic/sociodemographic variables. Generalized linear models were used to identify the association between the variables.

**Results:**

Of 1277 participants, 35.7% experienced high resilience. Poor sleep quality and sleepiness were present in 88.4% and 36.0% of students, respectively. High resilience was associated with good sleep quality (PR:1.56; 95%CI: 1.34 - 1.83; p-value<0.001), absent sleepiness (PR:1.59; 95%CI: 1.32–1.91; p-value<0.001), male sex (PR: 1.21; 95%CI: 1.05–1.39; p-value 0.006), working (PR:1.14), having family responsibilities (PR: 1.36; 95%CI: 1.09–1.70; p-value 0.005) and spending more than 6 hours studying (PR: 1.35; 95%CI: 1.17–1.54; p-value<0.001).

**Conclusion:**

4 out of 10 students presented high levels of resilience. The development of resilience depended on multiple individual and sociodemographic factors. These findings are important to support universities in developing resilience-building measures and strategies that can be implemented to mitigate the adverse pandemic event.

## Introduction

The coronavirus disease 2019 (COVID-19) was first identified in Wuhan, China, in December 2019, and on January 30, 2020, the World Health Organization (WHO) declared it as a global public health emergency (1). To curb its spread, most countries adopted exceptional measures such as mandatory confinement, quarantine, and social distancing (2), measures that generated negative effects in the fields of economy, health, and education worldwide (1). As for medical education, careers that for most years have been conducting face-to-face classes in hospitals and through contact with patients had to suspend all face-to-face activities and migrated to an online learning format with the modification of the university curriculum (3); but not all clinical courses could be taught since the skills necessary for medical performance (such as performing the physical examination) can only be learned with the patient (4).

It has been evidenced that stress is higher in medical students when the “curricular demand” is greater than the resources they have to overcome, levels that are higher compared to students in other careers (5, 6). In addition to presenting a higher risk of depression, anxiety, emotional exhaustion, and psychological distress are often experienced (7). Therefore, an optimal level of resilience is required to cope with the development of these deleterious effects on mental health. Resilience behaves as a psychological coping characteristic by effectively coping with acute and chronic stress (8), as well as having the ability to remain positive in the face of difficult and unexpected times such as the COVID-19 pandemic (9). Likewise, it is an essential aspect of well-being in medical education that enables students to bounce back from adversity and challenges (10).

Some studies addressed the emotional and cognitive correlates of distance education during pandemic confinement, investigating possible predictors of psychological distress and difficulties in academic performance, focusing, among others, on changes in study environment and learning concentration (11–14). In addition, medical students often have a higher risk of depression compared to students in other careers (15). The presence of depressive symptoms seems to occur already in the first year of the student’s medical training, especially in women (16, 17). It has also been found that medical students show strong problems associated with anxiety and depression, emotional distress, low perception of quality of life, problems related to alcohol consumption, and a propensity to use substances as cognitive enhancers. Moreover, distress represents one of the most important causes of empathy impairment. Learning to respond to the distress of others with well-regulated empathy is an essential developmental skill linked to positive health outcomes and medical professional skills (16–18).

Among the factors associated with resilience are good sleep quality and the absence of sleepiness, because they play a transcendental role in the general economy of the body by contributing to normal physiological and psychological functions (19). Conversely, poor sleep quality leads to two negative effects: a decrease in general alertness and slowing of cognitive processing (20). Among the studies found, the academic year, ethnicity, sex, parental education level, coping strategies, age, perceived good health, and non-use of medication are proposed as factors associated with the development of resilience (9, 21). However, these studies did not consider the influence of sleep quality, sleepiness, study time, number of courses completed, academic year completed, family responsibility, and work. Also, previous reports have been conducted in contexts other than Peru and those conducted here have been restricted to local universities and small sample size (8, 9, 21–23).

Few studies conducted during the pandemic have examined resilience and its associated factors in medical students. It has been shown that life satisfaction can be enhanced by resilience strategies, in which religion is an important factor (24). In the context of online education, resilience had a moderate mediating role in the association between stress and academic burnout (1, 25). On the other hand, the absence of direct support and the need to face daily responsibilities without family help can increase stress and reduce levels of resilience (26). The development of resilience was also related to the need to contribute to the health emergency, helping in hospitals, which was also a way to escape lockdown (27). Resilience behaviors during the pandemic, such as supporting social network, brain fitness, and finding meaning in life seem to be present in students that experienced post-traumatic growth (28). However, this evidence is still lacking in Peru since mental health research in absolute numbers is low (29), which raises the following research questions:

1) What is the level and prevalence of resilience in Peruvian medical students during the pandemic?
2) What are the educational variables and is sleep quality associated with resilience?

Therefore, this study aimed to identify the level of resilience and associated factors in Peruvian medical students during the second pandemic wave. The findings of this work will add to the current literature on resilience as a protective factor in the face of an unexpected event.

## Methods

### Study design and population

A multicenter, observational, cross-sectional study was conducted from November 19 to December 05, 2020, in 23 Peruvian medical schools. Students enrolled in at least 12 academic credits and who agreed to participate voluntarily by accepting informed consent were included. Students who were in their medical internship or last year of studies were excluded, as well as those who did not adequately complete the questionnaires. The sampling method was non-probabilistic.

### Procedure

To obtain a significant sample of students, a national online call was made to students interested in participating as authors of the study. This allowed the inclusion of students from different parts of the country. The authors from each region sought support for the surveys by selecting a representative from each participating university, who would be responsible for disseminating the questionnaire and informed consent virtually through the social networks most used among students (Facebook, Whatsapp, and Instagram). Additionally, the direct participation of students was requested to increase the sample. Data collection was constantly monitored using reminders on the online platforms and individually to potential participants. The survey was elaborated in Google Forms. Students entering the survey were presented with the first sheet of paper showing questions related to the selection criteria. Students who did not meet these criteria were sent to a single page where they were thanked for their participation. Students who met the selection criteria were sent an informed consent form in which information related to the study was provided and their voluntary confirmation to participate in the study was requested. In this way, a sample of 1277 participants was obtained.

### Questionnaire

The first section inquired about sex, age, marital status, education of the head of household, family members with whom he/she lives, resilience zone, year of study, family responsibility, state support, work while studying, work for his/her studies, number of subjects, time spent studying, current cycle status and study methods.

#### Dependent variable

Resilience - Abbreviated Connor-Davidson Scale (CD-RISC): of 10 items evaluated through a Likert scale with 5 options, which has been previously validated yielding a Cronbach’s α coefficient of 0.87 in a multi-occupational sample (30), very good results have been obtained on the psychometric properties using three samples of undergraduate students so its measurement is efficient for resilience (31). The result was categorized as determined elsewhere (31), using the cut-off point of 33, where ≥ 33 resulted in obtaining high resilience and < 33 as a low resilience level.

#### Independent variable

Pittsburgh Sleep Quality Index (PSQI): it was used the Spanish version of the which presents 19 self-applied questions designed to measure 7 domains called component scores: subjective sleep quality, sleep latency, sleep duration, habitual sleep efficiency, sleep disturbances, sleep medication use, and daytime dysfunction. Component scores range from 0 (no difficulty) to 3 (severe difficulty) and, when summed, produce an overall score ranging from 0 to 21. Scores ≥ 5 indicate sleep disorders as previously suggested (32). Internal consistency was good showing a Cronbach’s α of 0.83 (32), with a diagnostic sensitivity of 89.6% and specificity of 86.5% (33).

Epworth Sleepiness Scale (ESS): This assessed daytime sleepiness or tendency to fall asleep specifically in 8 daily life situations using the, the overall score has a range from 0 to 24, and a score ≥ 10 is considered positive for sleepiness, as determined previously (34). The scale was validated in Latin America with a Cronbach’s α value of 0.85 (34), a diagnostic sensitivity of 61.65%, and a specificity of 82.77% (35).

### Statistical analysis

Categorical variables were described as frequencies and percentages, and continuous variables as mean values (standard deviation) in sex, age (years), marital status, religion, education of the head of household, with whom they live during the academic cycle, area of residence, year of study, family responsibility, receive state aid, work while studying, work for their studies, number of subjects taken, time dedicated to studying, current cycle status, study method, sleep quality, sleepiness and resilience. The variable age was not considered for the bivariate and multivariate analysis because, according to the literature, age does not behave as an influential factor in the development of resilience (36).

For the evaluation of normal distribution, we used the histogram and the evaluation of skewness and kurtosis, the variables did not follow a normal distribution. The chi-square test was used to determine the association of the variables according to groups or categories. For simple and multiple regression analysis to estimate prevalence ratios (PR), generalized linear models (GLM) with Poisson distribution were used, with 95% confidence intervals and statistical significance greater than 5%.

Survey data were organized in Microsoft Windows Excel ^®^ (licensed for computer use for analysis) and analyzed in Stata 16.1 (College Station, TX: StataCorp LL).

## Results

### General description of the population

Of 1277 participants, the mean age was 22 years (SD: 3.7) and 58.3% were women. Poor sleep quality was present in 88.4% of the students and drowsiness in 36.0%. High resilience was experienced by 35.7% of participants (**Table 1**).

**Table 1:** Characteristics of Peruvian human medicine students (n=1277).

Characteristics	n (%)
Sex	
Female	745 (58.3)
Male	524 (41.0)
I prefer not to say	8 (1.7)
Age (years)*	21.9 ± 3.7
Marital status	
Single	1,138 (89.1)
Married	14 (1.1)
Dating	102 (7.9)
Committed	23 (1.8)
Religion	
Catholic	773 (60.5)
Non-Catholic	226 (17.7)
No religion	278 (21.7)
Living with family	
Yes	1,102 (86.3)
No	175 (13.7)
Urban residence	
Yes	1,163 (91,0)
No	114 (8.9)
Academic year	
First	222 (18.0)
Second	226 (18.4)
Third	198 (16.1)
Fourth	263 (21.4)
Fifth	131 (10.6)
Sixth	188 (15.3)
Family responsibility	
No	1,179 (92.3)
Yes	98 (7.6)
Working	
No	1,043 (81.6)
Yes	234 (18.3)
Number of courses taken	
< 4	598 (46.8)
> 4	679 (53.1)
Study time (hours)	
1 – 4	350 (27.4)
4 – 6	374 (29.2)
6 – 8	369 (28.9)
8 - 12	184 (14.4)
Current cycle status	
Regular	1,191 (93.2)
No regular	86 (6.7)
Study method	
Reading & memory	381 (21.6)
Class notes & reading	863 (49.1)
Reading only	158 (8.9)
Class notes only	83 (4.7)
No specific method	214 (12.1)
Other	58 (3.3)
Sleep quality	
Good	147 (11.5)
Bad	1,130 (88.4)
Drowsiness	
Yes	460 (36.0)
No	817 (63.9)
Resilience	
Low	821 (64.2)
High	456 (35.7)

*Mean ± standard deviation.

When evaluating the determinants associated with high resilience, a difference was found according to sex, as males had 9% more frequency of high resilience than females (p=0.001), time spent studying greater than 6 hours (40. 6% vs 31.9%; p=0.001), family responsibility (48.9% vs 34.6%; p=0.004), good sleep quality (57.8% vs 32.8%; p<0.001), as well as no sleepiness (41.6% vs 25.2%; p<0.001) (**Table 2**).

**Table 2:** Bivariate analysis of the determinants associated with the resilience of medical students in Peru (n=1277).

Characteristics	Resilience		
	Low resilience	High resilience	p*
	n (%)	n (%)	
Sex			
Female	506 (67.9)	239 (32.0)	**0.001**
Male	308 (58.7)	216 (41.2)	
Marital status			
Single	741 (64.3)	411 (35.6)	0.943
Married/Committed	80 (64.0)	45 (36.0)	
Religion			
Catholic	509 (65.8)	264 (34.1)	0.923
Non-Catholic	141 (62.3)	85 (37.6)	
No religion	171 (61.5)	107 (38.4)	
Living with family			
Yes	707 (64.1)	395 (35.8)	0.800
No	114 (65.1)	61 (34.8)	
Urban residence			
Yes	746 (64.1)	417 (35.8)	0.726
No	75 (65.7)	39 (34.2)	
Academic year			
First	126 (56.7)	96 (43.2)	**0.001**
Second	161 (71.2)	65 (28.7)	
Third	139 (70.2)	59 (29.8)	
Fourth	163 (61.9)	100 (38.0)	
Fifth	94 (71.7)	37 (28.2)	
Sixth	110 (58.5)	78 (41.4)	
Family responsibility			
No	771 (65.3)	408 (34.6)	**0.004**
Yes	50 (51.0)	48 (48.9)	
Working			
No	685 (65.6)	358 (34.3)	**0.029**
Yes	136 (58.1)	98 (41.8)	
Number of courses taken			
< 4	396 (66.2)	202 (33.7)	0.177
> 4	425 (62.5)	254 (37.4)	
Current cycle status			
Regular	759 (63.7)	432 (36.2)	0.118
No regular	62 (72.0)	24 (27.9)	
Study time (hours)			
< 6	493 (68.0)	231 (31.9)	**0.001**
> 6	328 (59.3)	225 (40.6)	
Study method			0.163
Reading & memory	248 (65.0)	133 (34.9)	
Class notes & reading	371 (62.6)	221 (37.3)	
Reading only	55 (59.7)	37 (40.2)	
Class notes only	26 (78.7)	7 (21.2)	
No specific study method	110 (69.6)	48 (30.3)	
Other	11 (52.3)	10 (47.6)	
Sleep quality			
Good	62 (42.1)	85 (57.8)	**< 0.001**
Bad	759 (67.1)	371 (32.8)	
Drowsiness			
No	477 (58.3)	340 (41.6)	**< 0.001**
Yes	344 (74.7)	116 (25.2)	

*P-value of categorical variables calculated with the chi-square test.

Bold values indicate statistically significant differences.

In the multivariate analysis, we found the probability of developing high resilience in different factors. In good sleepers, the probability of having high resilience was 1.56 times the probability of developing high resilience of poor sleepers (PR: 1.56; 95%CI: 1.34–1.83; p-value<0.001), in those who did not present daytime sleepiness vs. students with daytime sleepiness (PR: 1.59; 95%CI: 1.32–1.91; p-value<0.001). Regarding the sociodemographic variables being male (PR: 1.21; 95%CI: 1.05–1.39; p-value 0.006), having family responsibilities (PR: 1.36; 95%CI: 1.09–1.70; p-value 0.005), and dedicating more than six hours to study (PR: 1.35; 95%CI: 1.17–1.54; p-value<0.001), these results were statistically significant (**Table 3**).

**Table 3:** Characteristics associated with high resilience in Peruvian human medicine students, in simple and multiple regression analysis (n=1277).

Characteristics	High resilience					
	Simple regression		p*	Multiple regression		p*
	PR	95% CI		PR	95% CI	
Sex						
Female	Ref.			Ref.		
Male	1.28	1.06 - 1.54	**0.008**	1.21	1.05 - 1.39	**0.006**
Marital status						
Single	Ref.			Ref.		
Other	1.00	0,74 - 1.37	0.954	0.95	0.76 - 1.20	0.715
Education of household head						
No higher	Ref.			Ref.		
Higher	1.03	0.82 - 1.30	0.762	1.14	0.95 - 1.36	0.144
Living with family						
No	Ref.			Ref.		
Yes	1.02	0.78 - 1.34	0.839	0.91	0.73 - 1.13	0.417
Urban residence						
No	Ref.			Ref.		
Yes	1.04	0.75 - 1.45	0.779	0.97	0.76 - 1.23	0.715
Academic year						
First	Ref.			Ref.		
Second	0.66	0.48 - 0.91	**0.011**	0.70	0.55 - 0.89	**0.003**
Third	0.68	0.49 - 0.95	**0.024**	0.72	0.56 - 0.92	**0.011**
Fourth	0.87	0.66 - 1.16	0.368	0.93	0.74 - 1.16	0.538
Fifth	0.65	0.44 - 0.95	**0.028**	0.70	0.52 - 0.95	**0.022**
Sixth	0.95	0.71 - 1.29	0.786	0.99	0.79 - 1.25	0.998
Family responsibility						
No	Ref.			Ref.		
Yes	1.41	1.04 - 1.90	**0.023**	1.36	1.09 - 1.70	**0.005**
Number of courses taken						
< 4	Ref.			Ref.		
> 4	1.10	0.92 - 1.33	0.279	1.21	1.02 - 1.43	**0.021**
Current cycle status						
No regular	Ref.			Ref.		
Regular	1.29	0.86 - 1.96	0.211	0.97	0.71 - 1.31	0.846
Working						
No	Ref.			Ref.		
Yes	1.22	0.97 - 1.52	0.081	1.14	0.95 - 1.35	0.140
Study time (hours)						
< 6	Ref.			Ref.		
> 6	1.27	1.06 - 1.53	**0.009**	1.35	1.17 - 1.54	**<0.001**
Sleep quality						
Bad	Ref.			Ref.		
Good	1.76	1.39 - 2.22	**<0.001**	1.56	1.34 - 1.83	**<0.001**
Drowsiness						
No	Ref.			Ref.		
Yes	1.65	1.33 - 2.03	**<0.001**	1.59	1.32 - 1.91	**<0.001**

*P-values obtained using generalized linear models with Poisson distribution, log link function, and robust variance.

Bold values indicate statistically significant differences.

Ref, Reference value.

## Discussion

### Prevalence and level of resilience

It was found that 35.7% were found to have high resilience, and of these were associated with male sex, family responsibility, more than six hours to study, good sleeping, and no daytime sleepiness. This finding is similar to a study conducted in five medical schools in the United States, where they reported that 36.6% of students were resilient (37). While in South Africa, high levels of resilience were found (9). In this context, it is discouraging that more than 50% of students have low levels of resilience, although it is likely that the context of the COVID-19 pandemic has influenced the reduction of resilience to stress and academic demands in medical education so that medical students appear to be a particularly at-risk group. Evidence on interventions designed to improve individual resilience presents programs based on cognitive-behavioral therapy, mindfulness, or mixed interventions, which combine cognitive-behavioral therapy and mindfulness training. These resilience interventions based on a combination of cognitive-behavioral therapy and mindfulness techniques have shown a positive impact on individual resilience (38). Moreover, Padesky and Mooney’s strengths-based cognitive-behavioral therapy model is a useful alternative for enhancing resilience (39). Even so, computerized cognitive-behavioral therapy appears to be as effective a therapeutic strategy as face-to-face cognitive-behavioral therapy in the treatment of anxiety disorders (40).

In our study it was found that medical students belonging to the male sex developed greater resilience, similar results were found in students in the USA, Canada, and Turkey (41–43). This may be because women recognize and share their difficulties with the environment (44), have a worse response to stress, and are very self-critical; in contrast, men adopt coping strategies such as depersonalization to cope with stressful situations (45). The existing literature indicates that men, younger participants, single people, participants with higher education, and employed participants had higher levels of resilience. Women often take on additional roles in caring for the family and home, which increases stress and reduces the time available for self-care and emotional recovery, which can significantly impact their ability to develop resilience (46, 47). We found an association between high levels of resilience and working while studying. However, it was diluted in the adjusted regression. This association could be posited because burdensome experiences contribute to the development of resilience in healthcare students (48). Other factors could be the work environment and cross-cultural comparisons (49). Influencing the development of resilience.

Students who reported having family responsibility increased their level of resilience by 37.0%, no studies were found with direct associations between both variables since medical students are mostly young, single, and childless (50, 51), therefore they do not have significant family responsibility. Family responsibility can be observed mostly in married people and with children. In a study of university students, greater resilience was found in singles and fewer children of the participants (52). However, this association presented in our study could be because, as the family is the first place where we develop integrally, it favors the state of resilience, so we could infer that people who have family responsibility are more capable of facing problems and reacting to various changes, a result supported by a study that showed that family function improved resilience by 15.8% in medical interns (50).

Those students who dedicated more time to studying (> 6 hours) increased their resilience levels by 34.0%. No antecedents were found associating study time with resilience. However, study time is an indicator of academic performance (53). Thus, Chisholm Burns et al. found that resilience is generally associated with better academic performance outcomes (54). Similarly, Tempski et al. reported that the feeling of lack of time negatively impacted students’ quality of life (55), impairing the development of their resilience. This association could be due to the security that medical students have in dedicating more time to study, which predisposes them to better academic performance, allows them to cope with difficulties in a better way, and thus increase their resilience.

We found that students in the second, third, and fifth academic years presented lower levels of resilience at 30.0%, 28.0%, and 30.0%, respectively. This is consistent with Forycka et. al, who reported different levels of resilience in the 1st and 6th years than in others years (23), and Garayar et. al, who found higher resilience in students in the upper academic years (56). This association could be because first-year students suffer changes in their lifestyle when starting a demanding university career, on the other hand, in 6th year the academic workload of medical students towards the end of their career increases, which predisposes them to more development of resilience. This would indicate that more time of experience in a field can help increase resilience (57), first-year students may have started with good resilience but over the course of university due to complexity and anxiety, they fail to develop enough, and already in the final years this increases. Likewise, students who took more than four courses presented higher levels of resilience. This is consistent with Kiziela et al. who identified that this association could be due to students experiencing high levels of distress in the face of increasing academic demands placed on them, such as at the time of the pandemic, which forces them to adapt to these situations through the development of resilience (58). This association could be because the greater academic load leads to greater academic burnout and consequently lower levels of resilience (59). However, few studies report this association, so our study adds this result to the existing literature.

Students with good sleep quality had a 57.0% increase in the prevalence of resilience. This is consistent with Lenzo et al. who conducted research in the context of the pandemic, this association could be due to the presence of certain resilience factors such as “having something to live for”, mitigated the level of stress and depression during the period of COVID-19 contagion (60), and allowed for adequate sleep quality. It is noteworthy that this study had a different design and a different population. It is also consistent with Hrozanova et al. who determined that this association could be because to face the challenges imposed are addressed through the subcomponents of resilience such as social support that allows having social support that avoids experiencing stress and the structured style that favors adequate organization, to give adequate space to sleep (61), similarly it is consistent with Du et al. where this association could be because resilience decreases the effects of stress and anxiety caused by the pandemic on sleep quality (22), and is also consistent with Reis et al, who demonstrated that without difficulty falling asleep, there is a positive perception of academic performance and less anxiety, stress and higher levels of resilience (20). Not having sleepiness increased the prevalence of resilience by 58.0%. This association could be because students with a non-evening chronotype do not need to adapt to a morning or daytime schedule, since their circadian rhythm schedules are in agreement, and they do not present difficulties such as drowsiness at study times and present a better response to academic demands, consequently better academic performance and an adequate level of resilience (20). However, we recognize that exist an biderectional relation between the variables (62). The bidirectional relationship between sleep and resilience can be explained because both affect individuals’ brains through similar mechanisms. Both sleep and psychological resilience share key neural networks and brain centers. A person’s ability to adapt, especially in the face of intense stressful situations, is associated with the regulation of the activity of the ventromedial prefrontal cortex. This area is also involved in the pathophysiological problems derived from sleep disturbances (62, 63). Additionally, other brain regions, such as those involved in autonomic arousal (such as the HPA axis and noradrenergic, serotonergic, and dopaminergic systems) and emotional regulation (such as the hippocampus and amygdala), are related to both sleep and resilience. For example, sleep problems can lead to overactivation of the amygdala, which in turn can affect the ability to resist or recover from stressors. In addition, resilient students tend to handle academic stress better, which can also influence their quality of sleep, although to a lesser extent (64, 65).

### Limitations and strengths

Our study has limitations. First, the cross-sectional design of the study prevents us from identifying a causal association between the variables studied. This limitation could also manifest as an inverse association between the level of resilience and the factors assessed. Second, self-reported data were used, so all the students’ responses may not have been true, generating a measurement bias. This limitation is also reinforced by the overall online data collection method, in which there is no direct contact with the respondent to adequately explain the questions. Third, the sampling method was non-probabilistic, so the prevalence outcome of this sample cannot be inferred with certainty to the study population. Fourth, common method variance may occur since the same questionnaire was used to evaluate the variables of interest. However, different scales were used for CD-RISC, PSQI, and ESS, which may reduce this bias. Therefore, the study findings should be interpreted with caution. Despite these limitations, the strengths are noteworthy. First, a large sample of students belonging to 23 universities was included, which represents almost 50% of universities in the country. The large sample size also reduces the risk of type II error. Second, we obtained an acceptable response rate, reducing the probability of non-response bias. Third, we used questionnaires validated in the Peruvian population, which allow the results to be comparable with those of other populations. Finally, multiple regression analysis reduces confounding bias, so that the exploratory model proposed in this study provides useful hypotheses to test more rigorously in future research.

### Relevance of findings in mental health

One study found that perceived stress mediated the relationship between life events and quality of life in university students, negatively affecting physical and psychological health, as well as social relationships and the environment. Although resilience did not moderate these effects, its importance is evident, since low levels of resilience can increase vulnerability to stress, negatively affecting general well-being and increasing the risk of anxiety and depression (66). Few studies conducted during the pandemic have examined resilience and its associated factors in medical students. It has been shown that life satisfaction can be enhanced by resilience strategies, in which religion is an important factor (24). In the context of online education, resilience had a moderate mediating role in the association between stress and academic burnout (1, 25). The development of resilience was also related to the need to contribute to the health emergency, helping in hospitals, which was also a way to escape lockdown (27). Resilience behaviors during the pandemic, such as supporting social network, brain fitness, and finding meaning in life seem to be present in students that experienced post-traumatic growth (28). However, this evidence is still lacking in Peru since mental health research in absolute numbers is low (29).

This study examined resilience and its associated factors in medical students during the COVID-19 pandemic and is one of the few published in Peru, where mental health research in absolute numbers is low. Therefore, the findings add to the current literature on resilience as a protective factor in the face of an unexpected event. Little-studied factors were considered, such as sleep quality, sleepiness, study time, number of courses completed, academic year completed, family responsibility, and work. The level of stress experienced by each student has been overwhelming, in such a situation the development of resilience proposes to adequately cope with mental health burdens. However, we emphasize that it is vitally important to support universities in the development of coping tips, through which strategies can be implemented to mitigate the adverse events of the pandemic. In addition, it should be recognized that medical students represent a vulnerable group requiring increased attention during and after the pandemic. Therefore, we recommend periodic assessments of resilience due to its fluctuating course, and post-pandemic studies will be imperative.

To increase resilience in medical students, the following strategies can be adopted, Stress Management, through techniques such as meditation, mindfulness and relaxation exercises (67). Social support among peer support networks and mentoring programs, Time Management that offers workshops on effective time management to balance academic and personal life. and develop Resilience Training programs focused on Implement programs focused on building resilience, including coping strategies and positive thinking (67–69).

## Conclusions

We identified that four out of 10 students presented high levels of resilience. Among the factors associated with the highest level of resilience were male sex, working while studying, having family responsibilities, dedicating more than 6 hours to study, taking more than four courses, second to fourth academic year, good sleep quality, and absence of drowsiness.

## Data Availability

The raw data supporting the conclusions of this article will be made available by the authors, without undue reservation.
